# Breastfeeding, Human Milk and COVID-19—What Does the Evidence Say?

**DOI:** 10.3389/fped.2020.613339

**Published:** 2020-11-20

**Authors:** Leon R. Mitoulas, Nania G. Schärer-Hernández, Severine Liabat

**Affiliations:** ^1^Medela AG, Baar, Switzerland; ^2^Honorary Research Fellow, School of Molecular Sciences, The University of Western Australia, Perth, WA, Australia

**Keywords:** human milk, breast milk, breastfeeding, coronavirus, COVID-19, SARS-CoV-2, transmission, evidence

## Introduction

Severe acute respiratory syndrome coronavirus 2 (SARS-Cov-2), the virus responsible for COVID-19, is a recently discovered coronavirus that as of October 2020 has reached across the globe infecting over 33 million people, of which approximately 1 million have died (1). Coronaviruses are a large family of viruses known to cause respiratory infections in humans. These viruses also include Middle East Respiratory Syndrome (MERS) and Severe Acute Respiratory Syndrome (SARS).

The most common symptoms associated with COVID-19 are fever, dry cough, and tiredness. Other less common symptoms include nasal congestion, headache, conjunctivitis, sore throat, diarrhea, loss of taste or smell and general aches and pains. Importantly, some people may experience very mild symptoms whereas others, especially those with existing underlying medical problems, e.g., high blood pressure, heart and lung problems, diabetes, or cancer, are at a higher risk of serious illness and potentially death (2).

As the pandemic unfolded, many questions were asked with respect to transmission routes and modes of infection, with particular interest devoted to the mother-infant dyad and the status of breastfeeding and human milk. Whilst the benefits of human milk and breastfeeding to the mother and the infant are well-documented (3) there was uncertainty in the early stages of the pandemic with respect to hospital practices and recommendations. What was clear, however, was the need for evidence-based recommendations addressing the issue of transmission whilst accounting for the value of breastfeeding. Such recommendations would enable new mothers and their babies to continue benefitting from the advantages of breastfeeding and the use of human milk in this COVID-19 era.

## Horizontal Transmission

The primary mode of transmission of SARS-CoV-2 is horizontal transmission via direct, indirect, or close contact with infected people through secretions, such as saliva or respiratory droplets, expelled during talking, coughing, and/or sneezing (4). These respiratory droplets or aerosols may be the source of transmission via either airborne routes or from surface contact. Whilst airborne routes of transmission might be limited (5), aerosols and droplets can remain viable on surfaces such as cardboard, plastic, and stainless steel for up to 72 h (6).

Of particular interest is the ability to transmit the virus via other biological fluids. Active virus has been found in fecal samples and evidence of viral RNA has been observed in blood (7). Furthermore, viral RNA has been isolated from urine (8). However, it must be noted that to date there have been no reports of transmission via feces (9, 10) or urine (4) or via blood transfusion (9, 10).

## Vertical Transmission

The transmission of SARS-CoV-2 from mother to infant, vertical transmission, has been the source of intense scrutiny, and can be categorized as intrauterine/congenital, intrapartum, or postnatal (11, 12). To date, reports of intrauterine, or transplacental transmission have been described (13, 14), although cases in the literature are limited. Bwire et al. (15) summarized the findings from 205 infants born to mothers positive for COVID-19 and found that 6.3% of infants were infected. Transmission was reported for term and preterm infants and for vaginal and c-section modes of delivery. These data were supported by other reviews which concluded that neonatal COVID-19 infection is uncommon, almost never symptomatic, and that the rate of infection is no greater when the baby is born vaginally, breastfed, or allowed contact with the mother (16, 17).

Studies on postnatal transmission have focused on accepted contact (horizontal) routes as well as the investigation of vertical routes e.g., via human milk. Whereas, the horizontal transmission routes are well-accepted and characterized, the role that human milk plays, if at all, is still not fully elucidated.

To date, in the literature, there are no confirmed reports of postnatal transmission of SARS-CoV-2 via human milk. Whereas, a recent report did not detect any SARS-CoV-2 in milk samples collected from women with mild to moderate COVID-19 symptoms (18), some earlier reports in the literature have noted the presence of SARS-CoV-2 viral RNA in human milk (19–25). However, in none of these cases there was evidence that the virus was complete and/or active. It is also of note that only random milk samples were shown to be positive for viral RNA. For example, in the study conducted by Chambers et al. (23) only one milk sample from one mother on 1 day was positive. Milk samples from the same mother 2 days prior and 12 days later were negative. Similar transient positive identification of viral RNA in human milk has also been observed in other studies (20, 24). As such, the researchers could not rule out contamination of the milk via respiratory droplets from the mother as a possible explanation for the detection of viral RNA in the breast milk samples analyzed. Whilst continued research is required to better characterize the presence of the virus in human milk, current evidence suggests that human milk does not appear to play a role in the transmission of SARS-CoV-2 (26, 27).

Despite the limited reports documenting the presence of viral RNA in human milk and the lack of any data showing the presence of active virus, several studies have investigated the ability of pasteurization techniques to destroy the virus. All have shown that Holder Pasteurization (62.5°C for 30 min) is able to inactivate replication-competent SARS-CoV-2 virus that had been added into human milk samples in the laboratory (23, 28, 29). As Holder Pasteurization is the main method employed by milk banks, it confirms that Pasteurization of human milk is a safe and feasible option if the clinician or mother are in any doubt with respect to the continued feeding of the infant with human milk upon a positive SARS-CoV-2 infection of the mother.

## Discussion

As the evidence currently suggests, there is limited risk that SARS-CoV-2 can be transmitted via human milk. Consequently, the WHO (30) and other organizations [e.g., UNICEF (31), CDC (32), Royal College of Obstetricians and Gynaecologists (33)] recommend that mothers continue to breastfeed their infants. Indeed, the main concern in this instance is the mother's ability to follow the strict contact precautions to avoid spreading the virus via the recognized horizontal routes. Therefore, it is important that mothers be counseled on suitable protocols for breastfeeding and breast pump usage (12, 17, 26, 32).

The importance of the continuity of breastfeeding for the infant in these times cannot be overstated. In addition to the already well-known and documented benefits of breastfeeding (3), recent research has shown specific benefits relevant to the COVID-19 situation. For example, antibodies to SARS-CoV-2 have recently been isolated in human milk (34–36) and have shown strong SARS-CoV-2 neutralizing capabilities (18, 37). Whilst these data show a robust immunological response by human milk against the virus they also suggest that human milk provides an active form of protection against the virus, a form of protection that cannot be provided to the infant via artificial formula, thus further supporting the need for the protection and promotion of breastfeeding.

Despite the many general benefits of breastfeeding to the infant, as well as the potential COVID-19 specific immunological benefits, recent research has also highlighted the need to be aware of maternal mental health in relation to the COVID-19 pandemic. A survey of over 5,000 women in Belgium, showed an increased likelihood of anxiety among pregnant women and early postpartum mothers during the recent COVID-19 lockdown (38). Similar outcomes were observed in Italy, which resulted in a worsening of maternal depressive symptoms (39). Importantly, these data were supported by a smaller cohort of mothers in Canada, suggesting that this response is not merely cultural (40). It is well-known that increases in maternal depressive symptoms lead to decreases in breastfeeding confidence and self-efficacy, resulting in reduced breastfeeding duration (41, 42). Therefore, it will be important to anticipate these mental health issues for new mothers in this COVID-19 era and provide them with the extra support needed so that their mental health well-being is ensured and that they and their infants continue to benefit from all the advantages afforded through breastfeeding (43).

## Conclusion

Despite the relatively short time the research community has had to investigate SARS-CoV-2 and its impact on breastfeeding and human milk, a tremendous volume of work has been conducted and published. These data show no evidence of active virus in human milk and suggest that vertical transmission via breast milk is very unlikely. Furthermore, there is very good evidence that antibodies present in human milk can neutralize SARS-CoV-2, suggesting a protective role of human milk against infection. These data all suggest that mothers and their infants should be kept together and breastfeeding should be promoted. Indeed, it is the avoidance of the established horizontal transmission routes between a mother and her infant that should be the focus of attention and education. Guidelines and practice recommendations based on these data should be developed in order to promote and protect breastfeeding and the use of human milk during these unprecedented times.

## Author Contributions

LM, NS-H, and SL conceptualized, reviewed, and edited the manuscript. LM drafted the initial manuscript. All authors contributed to the article and approved the submitted version.

## Conflict of Interest

LM, NS-H, and SL are employees of Medela AG, Switzerland.
